# Cerebrospinal fluid mechanics across CNS barriers: from production, circulation, and clearance to mechanomedicine

**DOI:** 10.3389/fnins.2026.1848071

**Published:** 2026-07-08

**Authors:** Xinglin Cheng, Bei Deng, Yannan Zhao, Hanguang Li, Tianen Zhan, Dezhong Peng

**Affiliations:** 1 School of Acupuncture and Tuina, Chengdu University of Traditional Chinese Medicine, Chengdu, Sichuan, China; 2 Sichuan Nursing Vocational College, Chengdu, Sichuan, China

**Keywords:** 4D flow MRI, blood–CSF barrier, cerebrospinal fluid, hydrocephalus, mechanotransduction

## Abstract

**Background:**

Cerebrospinal fluid (CSF) is often framed as a transport medium, yet its motion and pressure dynamics impose continuous mechanical loading on central nervous system (CNS) barrier and interface systems. These cues span scales—from cilia-scale near-wall shear to craniospinal compliance-driven pulsatility—and may shape barrier phenotypes, immune programs, and clearance efficiency. In simple terms, this review asks how abnormal CSF motion is converted into barrier dysfunction and disease-relevant outcomes.

**Main body:**

We synthesize evidence that CSF mechanics is spatially heterogeneous along the production-to-outflow axis and is sensed by specialized mechanotransduction modules in choroid plexus epithelium, ventricular ependyma, perivascular astrocytic endfeet/neurovascular unit, and meningeal outflow/lymphatic pathways. We discuss how shear, pulsatile forcing, and pressure–compliance relationships interact with mechanosensitive ion/transport channels, ciliary polarity, glycocalyx–ECM/FAK signaling, junctional remodeling, and nuclear mechanotransduction to regulate permeability and immune–metabolic states. We highlight quantitative toolkits, including low-velocity 4D flow MRI, phase-contrast MRI, waveform metrics, microfluidic barrier platforms, and computational modeling, that enable mapping of patient-relevant mechanics to cell-level exposures. Disease sections emphasize mechanical failure modes: oscillatory overload and multi-site CSF–barrier disruption in hydrocephalus; loss of effective pulsatile transfer and impaired perivascular exchange in neurodegeneration; age-related stiffening and altered mechanosensitivity across barriers; meningeal outflow dysfunction with neuroinflammatory amplification; and acute mechanical disruption after trauma.

**Conclusion:**

We propose a mechanomedicine framework for CSF–barrier coupling that prioritizes measurable mechanical exposures, interface-specific mechanosensors, and actionable endpoints, including barrier state, permeability, immune trafficking, and imaging-derived coupling metrics. This framework supports closed-loop translational pipelines linking human phenotyping to mechanistically calibrated models and may guide strategies that modulate CSF dynamics or target mechanotransduction nodes with quantifiable outcomes.

## 1 Introduction: CSF beyond transport—a mechanical regulator of CNS interfaces

Cerebrospinal fluid is produced predominantly at the choroid plexus (ChP), propagates through ventricles and subarachnoid spaces, exchanges with parenchymal and perivascular compartments, and exits via distributed outflow routes (Fame, 2025). Along this axis, CSF is not a steady conduit; it is driven by cardiac and respiratory cycles and constrained by craniospinal compliance (Khani et al., 2025). These dynamics establish a mechanical microenvironment that repetitively loads CNS barriers—blood–CSF barrier (BCSFB), ventricular ependyma, perivascular exchange sites at the neurovascular unit, and meningeal outflow structures. Experimental and clinical work on intracranial pulsatility emphasizes that changes in compliance and pressure/flow pulsations may be mechanistically linked to neurological states, particularly in hydrocephalus and traumatic injury contexts (Gholampour et al., 2023; Legé et al., 2024; Wagshul et al., 2011). From a mechanomedicine perspective, the key question is not merely whether CSF flow changes in disease, but which mechanical exposures (shear, pulsatility, pressure gradients, deformation) act on which barrier cell types, through which mechanosensors, to yield which measurable barrier phenotypes. This framing is increasingly feasible because advanced imaging can quantify low-velocity CSF fields and coupling between cerebral blood flow and periarterial CSF movement in humans (Vikner et al., 2025; Zimmermann et al., 2025).

Accordingly, we organize the review around three coupled exposure domains (near-wall shear, pulsatile forcing, and pressure–compliance–stiffness coupling) and map them onto CSF-facing interfaces [ChP/BCSFB, ependyma, neurovascular unit (NVU)-associated exchange sites, and meningeal outflow] to identify mechanosensors, intermediate phenotypes, and actionable endpoints for imaging-to-model-to-intervention pipelines. Several excellent reviews have summarized CSF flow, glymphatic clearance, and CSF outflow routes (Eide et al., 2026; Kelley and Thomas, 2023; Plog and Nedergaard, 2018); in contrast, the present review complements these works by emphasizing an exposure-to-sensor-to-endpoint framework that explains how defined CSF mechanical cues are sensed by CNS barriers and translated into measurable barrier phenotypes and potential intervention targets. Table 1 provides a compact exposure–sensor–endpoint map for CSF mechanomedicine. Clinically, this framework helps distinguish whether a CSF disorder is mainly driven by altered pressure, impaired pulsatile transfer, barrier vulnerability, or outflow failure.

**TABLE 1:** Interface-specific exposure–sensor–endpoint map for cerebrospinal fluid (CSF) mechanomedicine.

Interface	Dominant mechanical exposure	Interface-specific biology	Candidate sensor/intermediate	Actionable endpoints	Representative references
Choroid plexus epithelium/BCSFB	ICP waveform transmission; pressure/flow oscillation; cyclic deformation	Secretory epithelium with tight junctions; ion/water transport; immune signaling	Piezo1/TRPV4; ENaC-related transport; junction–cytoskeleton tension; epithelial cilia	TEER; tracer permeability; secretion/transport assays; tight-junction localization; cytokine markers	Hulme et al., 2022; Katada et al., 2025; Mao et al., 2025; Solár et al., 2020; Wang Q. et al., 2024
Ventricular ependyma	Cilia-scale shear; disturbed ventricular flow regime; cyclic wall loading	Multiciliated ventricular surface; PCP-dependent directional microflow; distinct from ChP/BCSFB	Motile cilia; PCP programs; glycocalyx; cytoskeletal remodeling	Ciliary beat frequency; basal body orientation; near-wall flow mapping; tracer mixing; glycocalyx integrity	Herlyng et al., 2025; Iida et al., 2025; Mao et al., 2025
NVU-associated perivascular pathways	Arterial pulsatility; perivascular pressure oscillation; altered effective pulsatile transfer	Multicellular exchange unit involving astrocytic endfeet, endothelium, basement membrane, BBB, and AQP4	Astrocyte/endothelial mechanosensing; AQP4 organization; ECM–integrin–FAK signaling	Blood-to-CSF coupling metrics; AQP4 polarity; BBB permeability; inflammatory markers; clearance-related readouts	Hill et al., 2025; Rowsthorn et al., 2023; Vikner et al., 2025
Meningeal outflow/meningeal lymphatics	Venous pressure; pressure gradients; meningeal stiffness; dural ECM	Clearance–immune interface linking CSF outflow, dural immune surveillance, and cervical lymph node drainage	Lymphatic mechanosensing; ECM stiffness; pressure-sensitive drainage responses	CSF efflux metrics; lymphatic morphology; cervical lymph node drainage; immune-cell trafficking; cytokine profiles	Hitpass Romero et al., 2025; Jacob et al., 2022; Jukkola et al., 2024; Shah et al., 2022
System-level modifier: aging/disease mechanical milieu	Vascular/tissue stiffening; altered craniospinal compliance; impaired buffering of pulsatility	Modifies exposure intensity and cellular mechanosensitivity across interfaces	FAK/YAP–TAZ; cytoskeletal tension; inflammatory priming; altered mechanosensor gain	CSF pulsatility/coupling measures plus vascular compliance, outflow, immune, and barrier biomarkers	Fatima et al., 2025; Hansen et al., 2024a; Herzog et al., 2025; Konig et al., 2025; Lacolley et al., 2025; Simon et al., 2022

BCSFB, blood–CSF barrier; BBB, blood–brain barrier; ChP, choroid plexus; ECM, extracellular matrix; ICP, intracranial pressure; NVU, neurovascular unit; PCP, planar cell polarity; TEER, trans-epithelial/endothelial electrical resistance.

## 2 Mechanical characteristics of CSF relevant to CNS barriers

**FIGURE 1:**
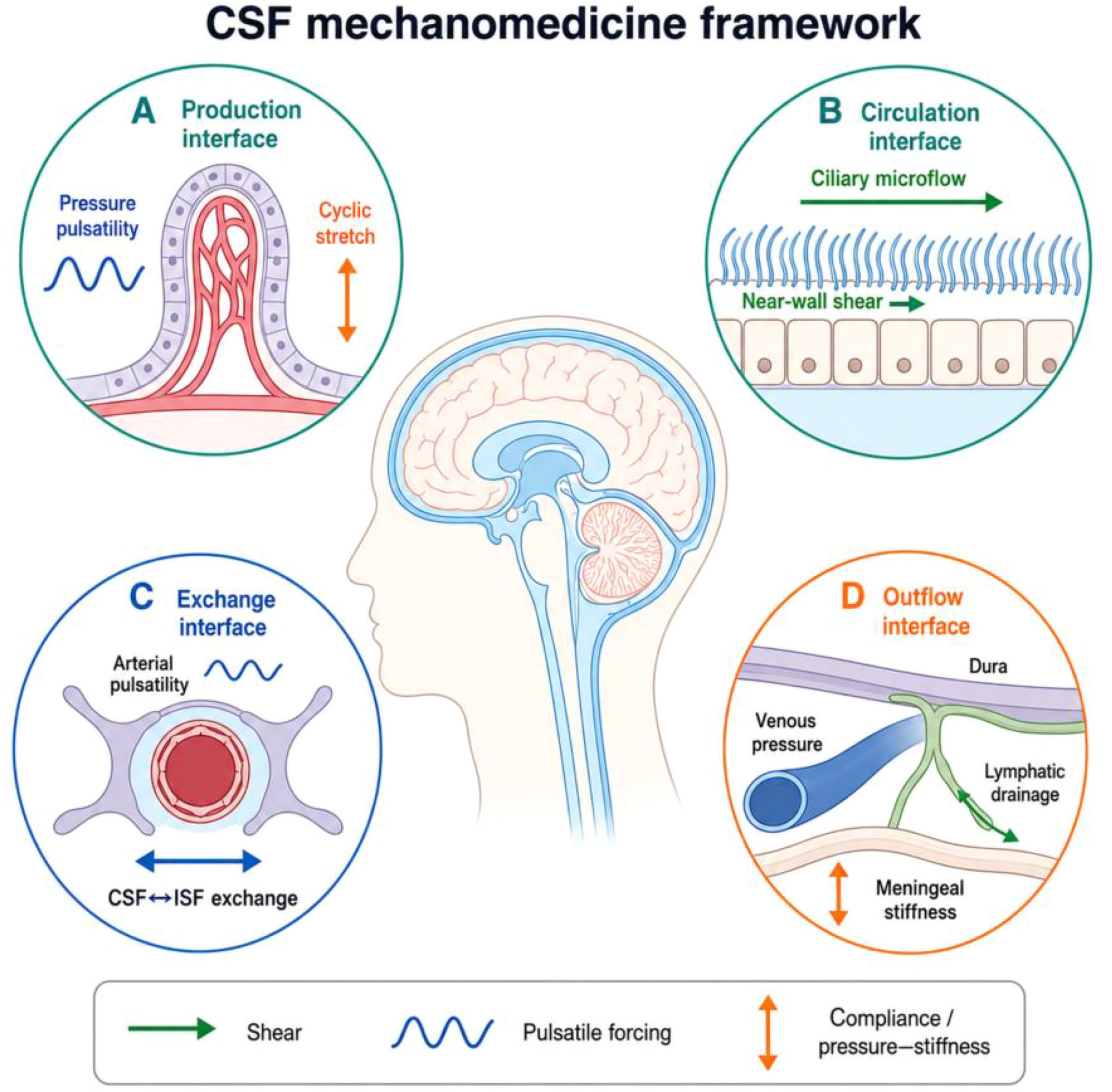
The cerebrospinal fluid (CSF) mechanomedicine framework along the production-to-outflow axis. This schematic summarizes four conceptual CSF-facing interfaces exposed to mechanical loading. **(A)** The production interface represents the choroid plexus/blood–CSF barrier, where pressure pulsatility and cyclic stretch may influence epithelial barrier and secretory functions. **(B)** The circulation interface represents the ventricular ependymal surface, where motile cilia generate ciliary microflow and local near-wall shear at the CSF-facing apical surface. **(C)** The exchange interface represents perivascular CSF–interstitial fluid (ISF) exchange coupled to arterial pulsatility. The bidirectional arrow denotes reciprocal CSF–ISF exchange rather than one-way bulk flow. **(D)** The outflow interface represents meningeal–lymphatic outflow, where venous pressure, lymphatic drainage, and meningeal stiffness may shape drainage efficiency. The insets are conceptual interface schematics rather than precise anatomical callouts to single points in the central brain illustration. Green arrows indicate shear-related cues, blue waveforms indicate pulsatile forcing, and orange double-headed arrows indicate compliance/pressure–stiffness-related loading.

Cerebrospinal fluid mechanics relevant to barrier biology can be organized into three coupled domains—near-wall shear, pulsatile forcing, and pressure–compliance–stiffness coupling. These variables are not independent: compliance shapes pulsatility transmission; pulsatility reshapes local shear histories; and local stiffness sets mechanotransduction gain. To orient subsequent sections, we summarize a mechanomedicine view of CSF–barrier coupling along the production-to-outflow axis, highlighting three coupled exposure domains—near-wall shear, pulsatile forcing, and compliance/pressure–stiffness relationships—and the interface sites where these exposures are sensed and converted into barrier phenotypes (Figure 1).

### 2.1 Shear stress at ventricular, subarachnoid, and meningeal surfaces

Barrier and interface cells primarily experience near-wall mechanical cues, which can differ substantially from bulk flow estimates (Katoh, 2023; Meng et al., 2022). In the ventricular system, ependymal motile cilia generate microflows and mixing, producing localized and spatially heterogeneous shear landscapes that may be more relevant to epithelial signaling than average ventricular velocities (Herlyng et al., 2025). In subarachnoid and meningeal compartments, CSF motion along dural and arachnoid surfaces generates low-magnitude but persistent shear (Leblond et al., 2024). Although such shear levels may be modest, they may still be biologically meaningful when mechanosensors have high sensitivity, when exposure is repetitive, or when inflammatory priming lowers activation thresholds (Hope et al., 2022).

Accordingly, claims about “shear stress at the ventricular or meningeal surface” should be made with explicit spatial-scale assumptions. By “compartment-level regime descriptors,” we refer to MRI-resolved measures such as bulk velocity, flow direction, pulsatility, and waveform features that characterize CSF motion at the scale of ventricles, subarachnoid spaces, or meningeal compartments, but do not directly specify the near-wall shear gradients experienced by epithelial, ependymal, or meningeal interface cells. Imaging-resolved velocities are therefore useful for classifying flow regimes and identifying altered CSF dynamics, but they should not be interpreted as direct measurements of cellular shear exposure without additional model-based or near-wall inference (Hurd et al., 2023; Vikner et al., 2024).

Near-wall shear depends on boundary-layer processes shaped by local geometry, wall motion, compliance, and, in the ventricles, cilia-driven microflows (Putluru et al., 2025). Mechanistic interpretations should therefore avoid equating bulk velocity magnitude with epithelial shear exposure and should distinguish qualitative associations, such as altered flow regimes, from quantitatively supported near-wall estimates. When quantitative inference is pursued, reporting should capture spatial and temporal heterogeneity, including oscillatory or direction-reversing components where relevant, because similar bulk velocity metrics can correspond to substantially different near-wall shear histories. For this reason, MRI-resolved velocities should be treated as compartment-level descriptors unless boundary-layer gradients are explicitly inferred using calibrated computational models, near-wall measurements, or experimentally controlled microfluidic systems.

### 2.2 Pulsatile forces in perivascular and barrier-adjacent spaces

Pulsatility is a defining feature of CSF dynamics and is driven by cardiac and respiratory cycles (Laganà et al., 2022; Wang et al., 2022). Arterial wall motion, venous volume changes, respiration-related pressure shifts, and intracranial pressure waveforms can transmit mechanical forces through periarterial spaces and other barrier-adjacent compartments, thereby shaping local CSF–interstitial fluid (ISF) exchange conditions (Gan et al., 2024). In humans, 4D flow MRI has provided in vivo evidence for coupling between cardiac-driven periarterial CSF motion and cerebral blood flow, offering quantifiable metrics that may link mechanical exposure to physiological exchange processes at perivascular interfaces (Vikner et al., 2025). These metrics are important for mechanomedicine because they allow pulsatile forcing to be measured in relation to CSF clearance, barrier state, and waste-removal hypotheses.

However, the transport consequences of pulsatility require careful terminology. Here, net advection refers to sustained one-directional transport, whereas pulsatile motion may mainly enhance back-and-forth exchange, mixing, or dispersion (Kelley and Thomas, 2023). Thus, a pulsatile waveform can enhance solute redistribution even when cycle-averaged displacement is small, and imaging or tracer findings should not automatically be interpreted as evidence of unidirectional bulk advection. This distinction is particularly important for perivascular exchange, where the magnitude and even the presence of net flow remain debated and may depend on anatomical scale, physiological state, and measurement approach (Eide et al., 2026; Kelley and Thomas, 2023; Lecchini-Visintini et al., 2025; Plog and Nedergaard, 2018).

Interpretation of tracer and imaging studies is also sensitive to experimental boundary conditions. Here, experimental boundary conditions include tracer infusion site, infusion volume and rate, tracer size and chemistry, anesthesia state, respiratory conditions, posture, pressure constraints, imaging time window, and, for computational models, assumed inlet/outlet conditions and wall-motion or compliance parameters (Zhu et al., 2023, 2026). These factors influence the apparent balance between diffusion, dispersion, reciprocal exchange, and net advection. Mechanomedicine studies should therefore report pulsatility metrics together with boundary conditions and should distinguish flow-regime changes from quantitatively demonstrated transport mechanisms.

### 2.3 Pressure gradients, compliance, and tissue stiffness across CNS compartments

Cerebrospinal fluid movement is driven by pressure gradients between CNS compartments, arising from cardiac/respiratory pulsations and regional production–absorption imbalance (Duy et al., 2024; Vinje et al., 2019). These gradients are strongly modulated by craniospinal compliance, including parenchymal, venous, meningeal, and spinal compliance, which governs how pulsatile pressure waves are buffered, propagated, and reflected across cranial and spinal CSF spaces (Putluru et al., 2025). In hydrocephalus or traumatic brain injury, reduced compliance may increase pressure pulse transmission and wave reflection, thereby altering transmural pressure, wall deformation, and shear-related exposures experienced by CSF-facing barrier interfaces (Hansen et al., 2024a; Saraiva et al., 2025).

For mechanomedicine, pressure–compliance–stiffness coupling should be treated as a measurable mechanical operating range rather than a single pressure value (Gholampour et al., 2023; Wagshul et al., 2011). These parameters can be viewed as clinical or experimental proxies of how strongly CSF pulsations are buffered or transmitted across CNS compartments. Relevant descriptors include intracranial pressure waveform features, pulse pressure or pulsatility indices, compliance or elastance proxies, pressure–volume relationships, transmantle or craniospinal pressure-gradient estimates, and vascular or tissue stiffness measures where available (Hansen et al., 2024a; Legé et al., 2024; Saraiva et al., 2025). Because these metrics vary with anatomical compartment, age, posture, disease state, acquisition method, and modeling assumptions, reported values should be interpreted as context-specific reference ranges rather than fixed physiological constants (Kelley and Thomas, 2023; Putluru et al., 2025). Representative literature-derived ranges for these CSF-relevant mechanical parameters are summarized in Table 2.

**TABLE 2:** Representative cerebrospinal fluid (CSF)-relevant mechanical parameters and translational interpretation.

Parameter	Representative range or value	Method or context	Mechanomedicine use	Key caveat	Representative references
Intracranial compliance	Approximately 0.45 mL/mmHg; 95% CI, 0.33–0.57 mL/mmHg	Infusion or pressure–volume analysis; hydrocephalus meta-analysis	Reflects craniospinal buffering capacity and sensitivity to pulsatile loading	Disease- and method-dependent; not a direct cellular exposure	Gholampour et al., 2023
Resistance to CSF outflow, rout	Approximately 14.93 mmHg/(mL/min); 95% CI, 13.65–16.21 mmHg/(mL/min)	Infusion testing; hydrocephalus/NPH cohorts	Defines outflow operating range and pressure-gradient loading	Does not specify local interface mechanics	Gholampour et al., 2023
Pressure–volume index, PVI	Approximately 19.26 mL; 95% CI, 15.63–22.89 mL	Pressure–volume analysis; hydrocephalus meta-analysis	Indicates pressure–volume reserve and compliance-related tolerance of volume change	Context-specific; not a universal physiological constant	Gholampour et al., 2023
ICP waveform features/pulse amplitude	No single universal range; interpreted using pulse amplitude, waveform morphology, posture, and disease state	Invasive ICP monitoring; waveform analysis; hydrocephalus, iNPH, trauma	Captures cyclic pressure loading and compliance failure affecting BCSFB, ependyma, and NVU	Requires acquisition context, anatomical compartment, and disease state	Gholampour et al., 2023; Legé et al., 2024; Wagshul et al., 2011
Aqueductal CSF stroke volume	Approximately 43–>200 μL per cardiac cycle in iNPH PC-MRI studies	Phase-contrast MRI; cerebral aqueduct	Compartment-level pulsatility and flow–volume coupling metric	Should not be equated with cilia-scale or near-wall epithelial shear	Whitley et al., 2024
Aqueductal peak CSF velocity	Approximately 5.9–12.8 cm/s in iNPH PC-MRI studies	Phase-contrast MRI; cerebral aqueduct	Describes pulsatile flow regime and supports patient stratification	Bulk velocity, not direct cellular shear exposure	Whitley et al., 2024
Ventricular CSF velocity/pulsatility metrics	Region- and subject-specific; reported as velocity, stroke volume, flow direction, pulsatility, and waveform features	Low-velocity 4D flow MRI; ventricular and craniospinal CSF spaces	Defines patient-specific boundary conditions for modeling and in vitro replication	Depends on acquisition settings, segmentation, temporal resolution, and anatomical region	Vikner et al., 2024, 2025
Respiratory modulation of CSF flow	Magnitude depends on breathing pattern, posture, anatomical region, and acquisition method	Real-time PC-MRI; ICP-based or flow-based modeling	Important boundary condition for pulsatility, pressure gradients, and CSF–barrier loading	Respiration and posture should be explicitly reported	Laganà et al., 2022; Vinje et al., 2019
Periarterial/perivascular pulsatile coupling	Quantified as coupling between vascular pulsation, cerebral blood flow, and periarterial CSF motion; method- and region-dependent	4D flow MRI and related human imaging approaches	Links vascular pulsatility to perivascular exchange and clearance-related hypotheses	Does not by itself prove net perivascular advection	Vikner et al., 2025; Zimmermann et al., 2025
Model-estimated near-wall shear	Study-specific; should be reported as model-derived ranges or sensitivity analyses	CFD/FSI; ventricular, spinal, subarachnoid, or meningeal models	Estimates cell-relevant shear not directly captured by MRI	Strongly depends on geometry, wall motion, compliance, and boundary conditions	Hurd et al., 2023; Katoh, 2023; Meng et al., 2022
Experimentally imposed shear/stretch	Operational low-to-moderate shear or stretch ranges selected according to the target cell type and barrier model	Microfluidic or barrier-on-chip platforms	Tests Ca^2 +^ signaling, TEER, permeability, junctional remodeling, and inflammation	Experimental range is not automatically physiological	Meng et al., 2022
Vascular or tissue stiffness descriptors	Region-, age-, and disease-dependent; reported using vascular stiffness indices, pulse-wave metrics, MRE, or ECM/hydrogel systems	Human imaging, vascular measures, MRE, or experimental matrix systems	Defines mechanical set-point and mechanotransduction gain at NVU, BCSFB, ependymal, and meningeal interfaces	Should be interpreted with age, vascular disease, anatomical region, and measurement method	Fatima et al., 2025; Herzog et al., 2025; Konig et al., 2025; Lacolley et al., 2025; Simon et al., 2022

Values are representative, context-dependent reference ranges rather than universal physiological constants. Imaging-derived velocity, stroke-volume, and pulsatility metrics should be interpreted as compartment-level descriptors unless near-wall shear or local deformation is inferred using calibrated computational models, near-wall measurements, or controlled experimental systems.

Tissue and vascular stiffness—shaped by aging and disease—further reshapes pressure–deformation relationships and the spatial distribution of mechanical stresses, potentially shifting mechanotransduction set-points and permeability regulation at the BCSFB, ventricular ependyma, and NVU-associated interfaces (Herzog et al., 2025; Konig et al., 2025; Lacolley et al., 2025). In neurodegeneration, increased vascular stiffness may weaken effective pulsatile transfer into perivascular routes, thereby compromising solute transport and waste clearance across the neurovascular unit (Fatima et al., 2025; Simon et al., 2022). Together, pressure gradients, compliance, and stiffness provide a mechanistic basis for understanding CSF-related disorders such as hydrocephalus, traumatic injury, and neurodegeneration, while also motivating standardized reporting of mechanical parameters for future exposure-to-phenotype mapping.

### 2.4 Quantifying CSF mechanics*: in vivo* imaging, *in vitro* platforms, and modeling

Quantifying CSF mechanics requires coordinated *in vivo* imaging, *in vitro* platforms, and computational models. In this workflow, imaging defines the patient-scale flow phenotype, modeling estimates difficult-to-measure local mechanical exposure, and *in vitro* platforms test causal cellular responses. Phase-contrast MRI and low-velocity 4D flow MRI enable visualization and quantification of CSF velocity fields, pulsatility, and flow–volume relationships in humans, including in hydrocephalus and idiopathic normal pressure hydrocephalus (iNPH) (El-Sayed Sakr et al., 2023; Karki et al., 2024; Vikner et al., 2024; Whitley et al., 2024). These imaging approaches provide compartment-level metrics such as velocity, stroke volume, flow direction, pulsatility, and waveform features, but they do not by themselves define the near-wall shear, local deformation, or cell-scale mechanical exposure experienced by CSF-facing barriers.

Microfluidic barrier platforms, organotypic cultures, and interface-specific in vitro systems provide controlled environments to impose defined shear, stretch, pressure, or pulsatile waveforms on relevant barrier cell types, enabling causal tests of mechanotransduction pathways under reproducible boundary conditions (Katoh, 2023; Meng et al., 2022). Such platforms are particularly useful when paired with barrier readouts such as trans-epithelial/endothelial electrical resistance (TEER), tracer permeability, calcium dynamics, junctional protein localization, cytoskeletal remodeling, and inflammatory markers. Computational and fluid–structure interaction models can integrate imaging and in vitro data to estimate difficult-to-measure exposures across compartments, including near-wall shear, local deformation, pressure gradients, and sensitivity to wall motion or compliance assumptions (Hurd et al., 2023; Putluru et al., 2025).

Representative literature-derived ranges for CSF-relevant mechanical parameters, including velocity/pulsatility metrics, pressure–compliance descriptors, model-estimated shear, and stiffness-related measures, are summarized in Table 2.

## 3 Barrier and interface systems exposed to CSF mechanics along the production-to-outflow axis

Cerebrospinal fluid-facing barriers are not mechanically equivalent along the production-to-outflow axis. Each interface is exposed to distinct combinations of shear, pulsatile forcing, pressure gradients, deformation, and stiffness, and each contains specialized cellular architectures that determine how mechanical inputs are converted into barrier phenotypes. This section therefore serves as a concise anatomical interface map, whereas mechanotransduction mechanisms and disease-specific failure modes are developed in sections “4 Mechanotransduction pathways linking CSF mechanics to barrier phenotypes” and “5 Mechanical failure modes of CSF–barrier coupling in disease,” respectively. The corresponding exposure–sensor–endpoint relationships are summarized in Table 1.

### 3.1 Choroid plexus and the blood–CSF barrier as the production interface

The choroid plexus (ChP) is the dominant CSF-producing tissue and forms the blood–CSF barrier (BCSFB) through tight junction–sealed epithelial layers, enabling selective transport of ions, nutrients, metabolites, and immune mediators from blood to CSF (Katada et al., 2025; Solár et al., 2020). In addition to biochemical and immune regulation, ChP epithelial cells experience intracranial pressure pulsatility, cyclic tissue deformation, and local flow-related cues. These mechanical inputs may influence epithelial transport, secretion, and barrier integrity through membrane, cytoskeletal, ciliary, and junction-associated mechanosensitive modules (Hulme et al., 2022; Mao et al., 2025).

Here, epithelial “transport programs” refer to ion, water, and solute transport pathways involved in CSF secretion and BCSFB function (Katada et al., 2025; Solár et al., 2020). Useful readouts include trans-epithelial electrical resistance (TEER), tracer permeability, tight-junction protein localization, transporter/channel expression or activity, secretion-related assays, and inflammatory cytokine markers (Robert et al., 2023; Wang Q. et al., 2024; Zhang et al., 2022). Importantly, ChP epithelium should not be treated as interchangeable with ventricular ependyma: ChP epithelial tight junctions support a selective BCSFB and secretory transport, whereas multiciliated ependymal cells primarily organize ventricular microflow and surface polarity (Herlyng et al., 2025; Mao et al., 2025; Solár et al., 2020). Thus, mechanical effects on ChP secretion and barrier integrity should be interpreted in a ChP-specific epithelial context.

In disorders with disturbed CSF dynamics, including hydrocephalus, the ChP can undergo functional remodeling; some settings show hypersecretory phenotypes that may aggravate ventricular enlargement (Robert et al., 2023; Zhang et al., 2022). In parallel, inflammatory activation at the ChP–BCSFB interface modulates tight-junction integrity and secretion-related transport pathways, positioning the ChP as both a transport and immune-regulatory interface (Wang Q. et al., 2024). A mechanomedicine agenda for the ChP therefore requires joint readouts of mechanical exposure, such as pressure/pulsatility and deformation, together with epithelial state, including transport activity, junctional integrity, and immune signaling.

### 3.2 Ventricular ependyma as a mechanosensory circulation interface

The ventricular ependyma lines the cerebral ventricles and forms a CSF-facing interface between ventricular fluid and periventricular tissue. Ependymal cells are specialized multiciliated cells whose coordinated motile cilia generate directional microflows and mixing, supporting ventricular transport and solute distribution (Herlyng et al., 2025). Because cilia and planar polarity programs are flow-responsive, the ependyma is positioned to convert local CSF mechanical cues into changes in ciliary beat coordination, surface organization, and barrier-adjacent signaling (Mao et al., 2025).

The ependymal interface differs from the ChP/BCSFB in both junctional organization and ciliary function. Whereas ChP epithelial tight junctions establish a secretory blood–CSF barrier, ependymal cells mainly provide a multiciliated ventricular surface that regulates microflow and exchange with periventricular tissue (Herlyng et al., 2025; Solár et al., 2020). Therefore, conclusions about cilia dynamics, junctional remodeling, or mechanosensitivity should not be generalized across ChP and ependyma without interface-specific evidence.

In hydrocephalus and other CSF flow disorders, altered flow regimes and pressure loading can disrupt ependymal integrity, ciliary function, and surface glycocalyx properties, contributing to impaired CSF circulation and altered CSF–tissue exchange (Iida et al., 2025). Mechanistic studies should distinguish direct effects of mechanical loading on ependymal mechanosensing from secondary injury pathways, such as inflammation or ventricular dilation. Practical readouts include ciliary beat frequency and coordination, basal body orientation, near-wall flow mapping, glycocalyx integrity, and junctional or permeability markers.

### 3.3 Perivascular astrocytic endfeet and the neurovascular unit at CSF–brain exchange sites

The neurovascular unit (NVU) is a major interface for CSF–brain exchange, particularly at perivascular spaces where CSF-adjacent fluid motion interacts with vascular pulsatility and interstitial fluid (ISF) transport. Astrocytic endfeet envelop cerebral vessels and interact with endothelial cells, pericytes, basement membrane components, and the blood–brain barrier (BBB) to regulate water and solute movement between perivascular compartments and brain parenchyma (Rowsthorn et al., 2023). Pulsatile blood flow and CSF pressure dynamics influence perivascular fluid motion and may contribute to waste-clearance processes (Han et al., 2024; Mortensen et al., 2025).

For the purpose of this interface map, we emphasize that the NVU is not a single barrier surface but a multicellular exchange unit (Hill et al., 2025; Rowsthorn et al., 2023). Relevant mechanical inputs include arterial wall pulsatility, perivascular pressure oscillations, local matrix stiffness, and altered pulsatile transfer into CSF–ISF exchange routes (Gan et al., 2024; Vikner et al., 2025). Corresponding readouts include imaging-derived pulsatility/coupling metrics, aquaporin-4 (AQP4) organization, astrocytic and endothelial mechanosensing markers, BBB permeability, and inflammatory state (Rowsthorn et al., 2023; Simon et al., 2022). Where evidence derives from rodent tracer or glymphatic studies, it should be interpreted as preclinical evidence and distinguished from human imaging-based observations (Eide et al., 2026; Zhu et al., 2023).

Astrocyte–endothelial communication at the NVU is molecularly specialized and context dependent (Hill et al., 2025). Disruptions in astrocyte state, perivascular organization, or channel localization can impair exchange and clearance, providing plausible links to neurodegeneration and vascular cognitive impairment (Cibelli et al., 2024; Huang H. et al., 2024). Mechanomedicine studies should therefore connect measurable pulsatility or coupling metrics to cell-state readouts under controlled inflammatory and vascular contexts, rather than treating reduced clearance as a purely hydrodynamic phenomenon.

### 3.4 Meningeal and lymphatic barriers shaping CSF outflow

Cerebrospinal fluid outflow has classically been associated with arachnoid granulations draining to dural venous sinuses (Shah et al., 2022). Additional meningeal lymphatic drainage circuits have been identified in mice and humans, expanding the anatomical and functional landscape of CSF clearance and immune surveillance (Jacob et al., 2022). These routes couple CSF outflow to dural immune surveillance by transporting solutes and immune cells from CSF-adjacent spaces toward cervical lymph nodes.

Mechanical factors—including venous pressure, meningeal stiffness, dural extracellular matrix remodeling, and pressure gradients across outflow routes—may influence drainage efficiency and lymphatic function, providing candidate mechanisms that link altered pressure environments or age-related tissue remodeling to impaired clearance and neuroinflammatory amplification (Hitpass Romero et al., 2025; Jukkola et al., 2024). Because much mechanistic evidence for meningeal lymphatic modulation remains preclinical, therapeutic extrapolation to humans should remain cautious.

A key mechanomedicine opportunity is to define how meningeal mechanical state sets the operating range of lymphatic drainage and immune trafficking. Useful endpoints include quantitative outflow metrics, dural or meningeal stiffness measures, lymphatic structural markers, cervical lymph node drainage, and paired immune readouts (Hitpass Romero et al., 2025; Jacob et al., 2022; Jukkola et al., 2024). These measurements can help determine whether outflow-targeted or pressure-modulating interventions produce measurable changes in clearance and inflammatory state.

## 4 Mechanotransduction pathways linking CSF mechanics to barrier phenotypes

**FIGURE 2:**
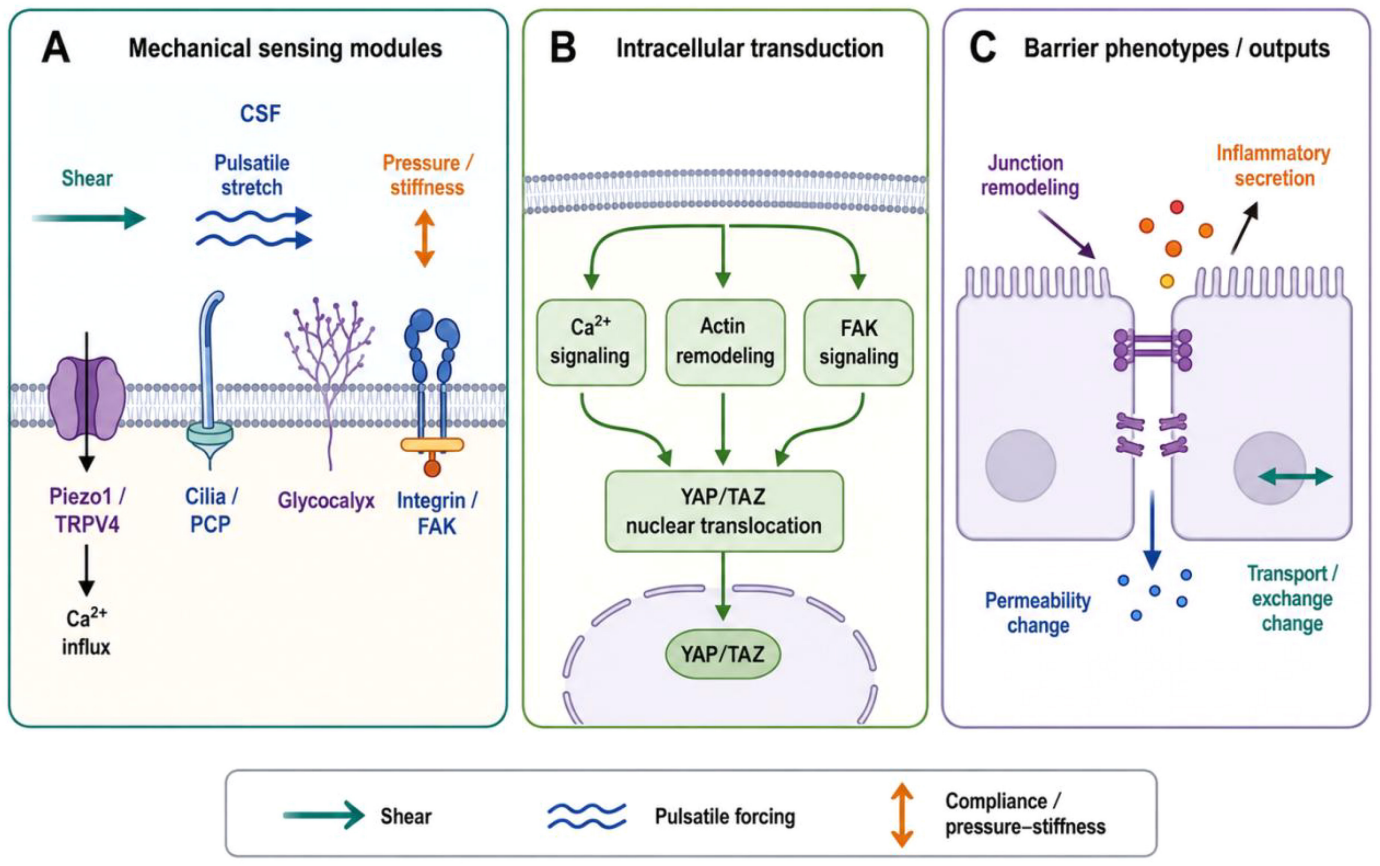
Mechanotransduction modules linking CSF mechanical cues to barrier phenotypes. **(A)** CSF-relevant mechanical cues, including shear, pulsatile forcing, and pressure/stiffness-related loading, may be sensed by mechanosensitive channels, cilia/PCP programs, the glycocalyx, and ECM-integrin-FAK signaling. **(B)** These sensing modules converge on intracellular transduction pathways, including Ca^2 +^ signaling, actin remodeling, FAK activation, and YAP/TAZ nuclear translocation. **(C)** These pathways lead to interface-specific barrier phenotypes and outputs, including junctional remodeling, permeability change, inflammatory secretion, and altered transport or exchange. Additional candidate channels, including TREK-1/TRAAK and ENaC, may contribute depending on cell type and disease context.

Cerebrospinal fluid dynamics generate shear, cyclic deformation, pressure fluctuations, and stiffness-dependent loading across CSF-exposed interfaces along the production-to-outflow axis. A mechanomedicine framework links these exposures to mechanotransduction modules and intermediate signaling layers that produce measurable barrier endpoints under defined physiological or pathological boundary conditions. Because ChP epithelium, ventricular ependyma, NVU-associated cells, and meningeal outflow structures differ in architecture and loading regimes, mechanotransduction mechanisms should be interpreted in an interface-specific manner rather than generalized across all CSF-facing barriers. The major mechanotransduction modules linking CSF mechanical cues to barrier phenotypes are summarized in Figure 2.

### 4.1 Mechanosensitive ion and transport channels in barrier and interface cells

These channels should be understood as molecular “mechanical translators” that convert stretch, shear, or pressure-related cues into calcium, ionic, inflammatory, or barrier responses. Across CSF-exposed interfaces and NVU-associated cells, Piezo1 and TRPV4 have been implicated as mechanically responsive Ca^2 +^-permeable channels that can reshape cytoskeletal tension, junctional organization, inflammatory programs, and barrier phenotype (Cibelli et al., 2024; Hochstetler et al., 2020). For causal inference, waveform-controlled loading should be paired with time-resolved Ca^2+^ readouts, barrier function assays and junctional protein dynamics as intermediate phenotypes.

Cerebrospinal fluid -interface mechanosensing, however, is not limited to Piezo1 and TRPV4. TREK-1 and TRAAK, members of the mechanosensitive two-pore-domain potassium channel family, can be activated by membrane tension and may tune membrane potential, ionic homeostasis, and mechanical responsiveness in loaded cells (Brohawn et al., 2014). ENaC-related sodium transport provides another candidate link between epithelial mechanical state and fluid/solute handling, because ENaC activity can be regulated by shear force in epithelial and vascular contexts (Baldin et al., 2020). At present, the extent to which TREK-1/TRAAK or ENaC operate as primary mechanosensors at ChP, ependymal, NVU, or meningeal interfaces remains insufficiently defined; therefore, these pathways should be interpreted as candidate or context-dependent modules unless validated in the relevant CSF-facing cell type.

Mechanical sensitivity is also strongly state dependent. In disease-relevant contexts, inflammatory tone can lower activation thresholds and amplify downstream responses to modest mechanical cues (Toft-Bertelsen et al., 2022; Yu et al., 2023). This predicts that mechanical dose–response relationships will vary across immune states and that mechanistic attribution is strongest when studies co-measure baseline inflammatory programs, channel activity, and barrier integrity alongside the applied mechanical exposure. *In vivo*, channel activity should therefore be interpreted as a composite of concurrent mechanical and biochemical inputs, rather than as a purely mechanical readout.

Together, these channel-level mechanisms provide testable entry points for CSF mechanomedicine. Candidate studies should pair defined shear, stretch, or pulsatile waveforms with channel-specific perturbation, Ca^2 +^ or electrophysiological readouts, TEER or permeability assays, junctional protein localization, and inflammatory markers. The major candidate mechanosensing modules and suggested readouts are summarized in Table 3.

**TABLE 3:** Candidate mechanosensing pathways at cerebrospinal fluid (CSF)-facing interfaces.

Mechanosensing module	Mechanical cue and relevant interface	Intermediate response and suggested readouts	Evidence note	Representative references
Piezo1/TRPV4	Shear, stretch, membrane tension, and pressure-related loading at ChP/BCSFB, ependymal, NVU-associated, endothelial, and meningeal interfaces	Ca^2 +^ influx; cytoskeletal tension; junctional remodeling; inflammatory activation; readouts include Ca^2 +^ dynamics, channel activity, TEER, tracer permeability, and junctional protein localization	Relatively well-supported mechanosensitive Ca^2 +^-permeable channels, but interface-specific roles require validation	Cibelli et al., 2024; Hochstetler et al., 2020; Yu et al., 2023
TREK-1/TRAAK K2P channels	Membrane tension, stretch, and lipid bilayer deformation in mechanically loaded barrier or interface cells	K^+^ conductance; membrane potential regulation; ionic homeostasis; readouts include expression, electrophysiology, and pharmacological or genetic modulation	Mechanosensitivity is supported in general systems; direct evidence at CSF-facing interfaces remains limited	Brohawn et al., 2014
ENaC-related sodium transport	Shear force and epithelial mechanical stimulation in epithelial or vascular contexts; candidate relevance to ChP/BCSFB and fluid-handling interfaces	Na^+^ transport; epithelial fluid handling; transporter activity; secretion-related assays	Candidate module linking mechanical stimulation to ion/fluid transport; CSF-interface validation remains needed	Baldin et al., 2020
Motile cilia/PCP programs	Cilia-scale shear, local flow direction, and disturbed microflow at the ventricular ependyma; ChP cilia should be interpreted separately	Ciliary beat frequency; ciliary coordination; basal body orientation; PCP markers; near-wall flow mapping; tracer mixing	Strong relevance for ependymal flow organization; avoid direct extrapolation to ChP epithelium	Bigotte et al., 2025; Bunatyan et al., 2023; Herlyng et al., 2025; Mao et al., 2025; Pan et al., 2024; Pellicciotta et al., 2020; Wallmeier et al., 2019
Glycocalyx	Near-wall shear and pressure/flow perturbation at ependymal, endothelial/NVU, and epithelial surfaces	Glycocalyx integrity; surface vulnerability; permeability change; inflammatory sensitivity	Plausible surface mechanosensing and protective layer; evidence strength varies by interface	Iida et al., 2025
ECM–integrin–FAK signaling	Matrix stiffness, deformation, and stretch at NVU, BCSFB, meningeal interfaces, and barrier-on-chip systems	FAK activation; focal adhesion organization; actin remodeling; junctional remodeling	Strong general mechanobiology rationale; CSF-specific evidence is emerging	Sun et al., 2016
Cytoskeletal–junctional remodeling	Shear, stretch, and cyclic deformation across ChP/BCSFB, ependyma, BBB/NVU, and meningeal barriers	Actin organization; tight/adherens junction localization; TEER; tracer permeability	Core intermediate linking mechanical loading to barrier function	Hollósi et al., 2021
YAP/TAZ nuclear mechanotransduction	Sustained stiffness, stretch, and cytoskeletal tension across CSF-facing interfaces	YAP/TAZ nuclear localization; transcriptional remodeling; repair/remodeling responses; inflammatory responsiveness	Downstream mechanotransduction integrator rather than primary sensor	Gong et al., 2021; Peng et al., 2025; Sun et al., 2024
Inflammatory and metabolic coupling	Mechanical loading under inflammatory priming or metabolic stress across all CSF-facing interfaces	Cytokines; adhesion molecules; immune trafficking; transporter/channel expression; metabolic stress markers; permeability assays	Modifies mechanosensor gain and barrier response; essential for causal interpretation in disease contexts	Hansen et al., 2024a; Huang X. et al., 2024; Jui et al., 2024; Yan et al., 2023; Yu et al., 2023

Evidence notes distinguish established mechanobiological mechanisms from candidate CSF-interface mechanisms. For TREK-1/TRAAK and ENaC, the table highlights plausible mechanosensing or transport-related modules that require further validation in specific CSF-facing cell types.

### 4.2 Cilia- and polarity-dependent sensing of CSF flow

At the ventricular surface, motile ependymal cilia sit at the interface between CSF motion and epithelial organization. Cilia density, local flow velocity, and alignment are tightly coupled: microflow patterns can reinforce coordinated beating and planar polarity, whereas disturbed flows can degrade coordination and reshape near-wall transport (Bigotte et al., 2025; Pellicciotta et al., 2020). Beyond driving CSF motion, ependymal cilia may also function as mechanosensory structures that encode local shear histories and help maintain tissue-scale directional transport (Pan et al., 2024).

When ependymal ciliary function is compromised—genetically or by altered CSF mechanics—ventricular transport efficiency can decline, with downstream consequences including impaired mixing, disrupted surface organization, and ventricular dilation (Pan et al., 2024; Wallmeier et al., 2019). Planar cell polarity (PCP) programs are central to this coordination, because basal body orientation and axonemal alignment determine the directionality and coherence of cilia-driven flow (Bunatyan et al., 2023). This module enables cross-scale readouts: ciliary beat frequency and coordination, basal body orientation distributions, near-wall flow mapping, and tracer mixing can be linked to ventricular transport outcomes under defined perturbations.

Cilia-related mechanosensing should be interpreted in an interface-specific manner. ChP epithelial cilia and ventricular ependymal motile cilia differ in cellular context, junctional organization, and dominant physiological role: ChP epithelial cilia are embedded in a secretory BCSFB epithelium and may participate in epithelial signaling or secretion-related regulation, whereas multiciliated ependymal cells primarily organize ventricular microflow and surface polarity (Herlyng et al., 2025; Mao et al., 2025). Therefore, observations about ciliary dynamics, polarity-dependent sensing, or junctional remodeling in one CSF-facing interface should not be directly extrapolated to another without matched cell-type and loading-regime evidence.

For translation, bulk imaging velocities should not be treated as direct surrogates for cilia-scale shear exposure. Instead, imaging-derived flow metrics should be framed as compartment-level flow-regime descriptors unless near-wall inference is explicitly supported by calibrated modeling, near-wall measurements, or experimentally controlled microfluidic/organotypic systems.

### 4.3 Glycocalyx–ECM coupling, cytoskeletal–junctional remodeling, and nuclear mechanotransduction

Conceptually, this section traces how mechanical forces are transmitted from the CSF-facing cell surface to junctions, the cytoskeleton, ECM adhesions, and the nucleus. At the cell surface, the glycocalyx may act as a hydrated mechanosensitive layer that filters and transmits shear-related forces to membrane, cytoskeletal, and junctional signaling pathways (Iida et al., 2025; Tarbell and Pahakis, 2006). In the ependymal compartment, age-dependent and post-intraventricular hemorrhage remodeling of the glycocalyx supports the concept that the CSF-facing surface layer may modulate interface vulnerability under altered flow or pressure states (Iida et al., 2025).

Extracellular matrix stiffness and matrix remodeling provide another mechanotransduction route. Through integrin engagement and focal adhesion kinase (FAK) activation, extracellular mechanical state can be coupled to actin tension, focal adhesion dynamics, junctional remodeling, and downstream nuclear signaling (Hollósi et al., 2021; Sun et al., 2016). This pathway is particularly relevant when aging, inflammation, hydrocephalus, or trauma alters matrix composition or stiffness, thereby shifting the mechanical set-point of CSF-facing barrier cells.

Shear and stretch can reorganize actin and adhesion complexes, driving dynamic remodeling of tight and adherens junctions with direct effects on barrier permeability (Du et al., 2024; Hollósi et al., 2021). Over longer timescales, nuclear mechanotransduction pathways —particularly Hippo-pathway mechanosensitive transcriptional co-activators YAP/TAZ—integrate mechanical context into transcriptional programs that influence differentiation, inflammatory responsiveness, and repair/remodeling capacity (Gong et al., 2021; Peng et al., 2025; Sun et al., 2024). In mechanomedicine terms, transient mechanical stimuli can be “written” into sustained barrier states through glycocalyx–cytoskeleton–junction coupling, ECM–integrin–FAK signaling, and nucleo-cytoskeletal mechanotransduction.

Accordingly, functional endpoints should extend beyond steady-state permeability to include junctional remodeling kinetics, tight-junction protein localization, cytoskeletal architecture under load, glycocalyx integrity, focal adhesion organization, FAK activation, and YAP/TAZ localization linked to transport and immune readouts. Because ChP epithelium, ventricular ependyma, NVU-associated cells, and meningeal interfaces differ in baseline architecture, surface specialization, ECM context, and loading regimes, cross-interface extrapolation should remain hypothesis-driven unless supported by interface-specific measurements.

### 4.4 Integration with inflammatory, metabolic, and permeability programs

Mechanical cues rarely operate in isolation. Instead, they enter state-dependent feedback loops that couple mechanosensing to immune activation, metabolic demand, and permeability regulation. Shear, stretch, deformation, and stiffness-dependent loading can modulate cytokine production, adhesion programs, immune cell trafficking, and junctional stability, while inflammatory mediators can in turn sensitize mechanosensitive channels, remodel the glycocalyx or ECM, and alter cytoskeletal–junctional tension (Hansen et al., 2024a; Jui et al., 2024; Yu et al., 2023). Thus, the same CSF mechanical disturbance may be harmless in a healthy barrier but damaging in an inflamed or metabolically stressed barrier.

Metabolic and transport programs provide another integration layer. In CSF-facing epithelia and NVU-associated cells, mechanical context may influence ion/water handling, transporter activity, mitochondrial demand, and energy-dependent junctional maintenance (Robert et al., 2023; Yan et al., 2023). Conversely, metabolic stress or inflammatory activation may reduce the capacity of barrier cells to maintain polarity, transport homeostasis, and permeability control under mechanical load. Thus, mechanotransduction should be interpreted as a coupled mechanical–immune–metabolic process rather than a purely biophysical response.

In disease settings, this coupling can become self-reinforcing. Mechanically triggered inflammation may increase barrier fragility, whereas barrier leakage or junctional disruption can alter local hydrodynamics, tissue stiffness, immune exposure, and mechanosensor gain (Huang X. et al., 2024; Jui et al., 2024; Robert et al., 2023). Such feedback loops are particularly relevant in hydrocephalus, aging, neurodegeneration, and trauma, where altered CSF mechanics and inflammatory injury may amplify each other over time.

Capturing these loops requires readout strategies that quantify mechanical exposure together with immune, metabolic, and permeability states (Hansen et al., 2024a; Jui et al., 2024; Robert et al., 2023). Useful endpoints include cytokine and chemokine profiles, adhesion molecules, immune-cell trafficking assays, transporter/channel expression, mitochondrial or metabolic stress markers, TEER, tracer permeability, junctional protein localization, glycocalyx integrity, FAK/YAP–TAZ activity, and imaging-derived CSF coupling metrics (Hollósi et al., 2021; Robert et al., 2023; Tarbell and Pahakis, 2006). For causal interpretation, studies should report baseline inflammatory tone, define the timing of mechanical versus immune perturbations, and distinguish primary mechanosensing from secondary injury responses.

## 5 Mechanical failure modes of CSF–barrier coupling in disease

**FIGURE 3:**
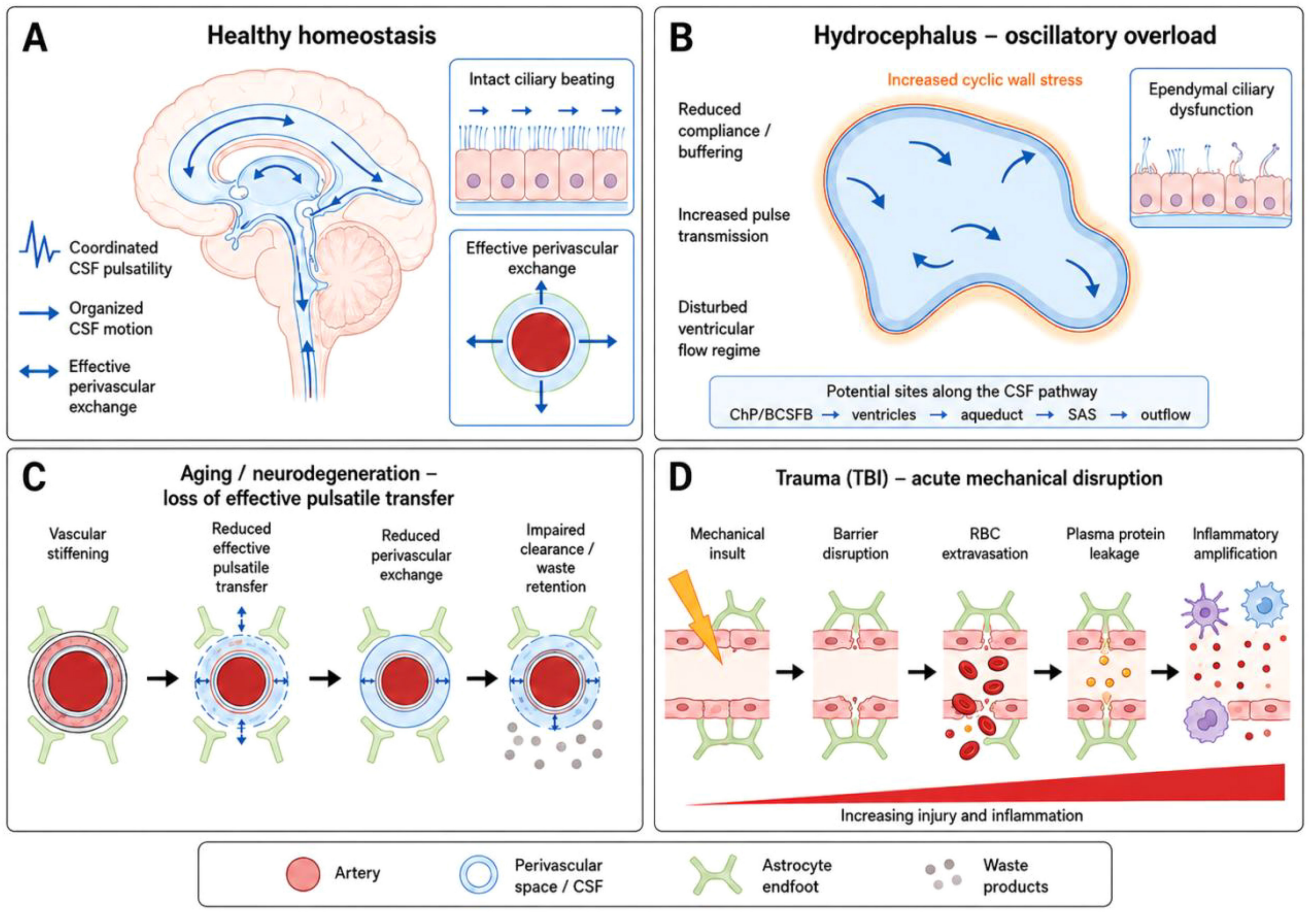
Disease-associated mechanical endotypes of CSF–barrier coupling. This schematic summarizes representative mechanical failure modes linking altered CSF mechanics to barrier and interface dysfunction in CNS disease. **(A)** In healthy homeostasis, coordinated CSF pulsatility and organized CSF motion support intact ependymal ciliary beating and effective perivascular exchange. **(B)** In hydrocephalus, reduced craniospinal compliance and impaired buffering may increase pulse transmission and cyclic wall stress at CSF-facing interfaces. The orange periventricular rim denotes increased cyclic wall stress, and the short internal arrows indicate a disturbed ventricular flow regime without implying a defined recirculating flow pattern. The enlarged ventricle is shown as a representative site of chronic mechanical loading; the route strip indicates that mechanical failure may involve multiple sites along the CSF pathway, including the choroid plexus/blood–CSF barrier, ventricles, aqueduct, subarachnoid space, and outflow routes. **(C)** In aging and neurodegeneration, vascular stiffening and altered compliance may reduce effective pulsatile transfer into perivascular spaces, leading to reduced perivascular exchange and impaired clearance or waste retention. **(D)** In traumatic brain injury, acute mechanical insult may disrupt barrier integrity, allowing red blood cell extravasation and plasma protein leakage, followed by inflammatory amplification. These panels illustrate dominant disease-associated mechanical endotypes and do not imply that pathology is restricted to a single anatomical site or mechanism. BCSFB, blood–CSF barrier; ChP, choroid plexus; CSF, cerebrospinal fluid; RBC, red blood cell; SAS, subarachnoid space; TBI, traumatic brain injury.

Different CNS disorders can be reframed as overlapping mechanical failure modes of CSF–barrier coupling, including oscillatory overload, loss of effective pulsatile transfer, compliance–stiffness imbalance, impaired outflow, and acute mechanical disruption. Rather than assigning a single CSF-flow abnormality to each disease, this section maps dominant mechanical exposures to vulnerable CSF-facing interfaces and measurable barrier endpoints in hydrocephalus, neurodegeneration, aging, meningeal outflow dysfunction, and trauma (Figure 3).

### 5.1 Hydrocephalus: oscillatory overload and multi-site CSF–barrier failure

Hydrocephalus reflects a breakdown of the CSF production–circulation–absorption/outflow axis, with ventricular enlargement and altered intracranial pressure (ICP) dynamics (Duy et al., 2024). Mechanical failure may occur at multiple points along this route, including ChP-mediated CSF production, lateral and third/fourth ventricular conduits, aqueductal or foraminal flow pathways, subarachnoid spaces, arachnoid granulations, venous drainage, and lymphatic outflow routes. Thus, the lateral ventricular wall should be viewed as a representative site of chronic mechanical loading rather than the exclusive locus of hydrocephalus pathophysiology.

From a mechanomedicine perspective, a useful organizing concept is oscillatory overload: abnormal waveform transmission and impaired craniospinal buffering increase cyclic mechanical stress on CSF-facing interfaces, including the BCSFB and ventricular lining (Duy et al., 2024; Murambi et al., 2025). This reframes hydrocephalus not only as a fluid accumulation disorder but also as a chronic loading disorder in which pressure pulsatility, local deformation, near-wall shear, and compliance failure can interact across the production-to-outflow pathway.

The ChP is central to this framework because it is both the major site of CSF production and a mechanosensitive epithelial barrier. In hydrocephalus, ChP epithelial remodeling, inflammatory activation, altered ion/water transport, and hypersecretory phenotypes may contribute to CSF dysregulation and ventricular enlargement (Robert et al., 2023; Wang Q. et al., 2024; Zhang et al., 2022). Therefore, hydrocephalus-related mechanical loading should be evaluated not only at the ependymal wall but also at the ChP/BCSFB, where pressure pulsatility, deformation, and inflammatory signaling may converge on secretion and barrier integrity.

At the ventricular surface, sustained cyclic loading can push the ependyma beyond resilience thresholds, contributing to ciliary impairment, loss of surface organization, glycocalyx remodeling, and junctional compromise (Iida et al., 2025; Visser et al., 2021). As these protective features erode, permeability may increase and CSF–tissue exchange may become dysregulated, potentially promoting periventricular injury and further coupling pressure dynamics to barrier dysfunction (Pang et al., 2025). In this setting, ependymal failure should be interpreted as one component of a broader mechanical failure network rather than as the sole driver of hydrocephalus.

Mechanical stress and inflammation can amplify each other. Ependymal and ChP responses may include inflammatory activation that destabilizes barrier phenotype and impairs CSF handling (Iida et al., 2025; Robert et al., 2023). Emerging work suggests that modulating pulsatility or intracranial pressure may improve barrier integrity and CSF circulation in selected hydrocephalus contexts (Choi et al., 2024). However, therapeutic interpretation should remain tied to measurable exposure changes and barrier endpoints rather than assuming that any intervention that changes bulk CSF flow necessarily normalizes cellular mechanical loading.

Future work should quantify hydrocephalus-relevant exposures across the CSF route, including ICP waveform features, local deformation, near-wall shear, compliance proxies, and production/outflow-related metrics. These should be linked to interface-specific phenotypes such as ChP secretion and inflammatory state, BCSFB junctional integrity, ependymal ciliary coordination, glycocalyx integrity, permeability, and periventricular injury markers. Such exposure-to-phenotype mapping is essential for stratification and for selecting mechanically meaningful endpoints for therapy development.

### 5.2 Neurodegeneration: loss of effective pulsatility and impaired perivascular exchange/clearance

In neurodegenerative diseases, including Alzheimer’s disease, Parkinson’s disease, and vascular dementia, CSF dynamics and clearance-related pathways are frequently altered (Dagum et al., 2026; Huang H. et al., 2024; Li et al., 2022; Liang et al., 2023). Within a CSF mechanomedicine framework, a recurring mechanical signature is the loss of effective pulsatility: impaired transfer of cardiac- and respiratory-driven mechanical energy into periarterial/perivascular exchange routes. This does not simply mean that global CSF flow is reduced; rather, it refers to weakened coupling between vascular pulsation, CSF motion, and CSF–interstitial fluid (ISF) exchange at NVU-associated perivascular interfaces (Vikner et al., 2025; Wang et al., 2022; Zimmermann et al., 2025).

Several mechanisms may contribute to this loss of effective pulsatility. Vascular stiffening, altered craniospinal compliance, impaired respiratory–cardiac coupling, and age- or disease-related changes in vessel wall motion may blunt the transmission of pulsatile energy into perivascular spaces (Bancroft et al., 2025; Wen et al., 2022). Because perivascular pathways are central to glymphatic concepts and NVU-mediated exchange, reduced effective pulsatility may impair exchange, mixing, or clearance-related coupling without necessarily implying a large unidirectional bulk flow (Eide et al., 2026; Kelley and Thomas, 2023; Plog and Nedergaard, 2018; Rowsthorn et al., 2023). This distinction is important because the magnitude and even the presence of net perivascular advection remain debated and may depend on species, anatomical scale, physiological state, and measurement approach.

At the cellular interface, altered pulsatile transfer may interact with astrocytic endfeet, endothelial cells, basement membrane properties, and aquaporin-4 (AQP4) organization. Disrupted AQP4 localization and NVU dysfunction have been linked to impaired glymphatic exchange, protein aggregation, and neuroinflammation in preclinical and disease-associated contexts (Huang H. et al., 2024; Kritsilis et al., 2025; Simon et al., 2022). However, rodent tracer findings and human imaging observations should be interpreted separately: rodent studies provide mechanistic evidence, whereas human MRI studies more often provide indirect coupling or clearance-related metrics (Eide et al., 2026; Rowsthorn et al., 2023; Zhu et al., 2023).

Neuroinflammation may both result from and amplify impaired perivascular exchange. Reduced clearance-related coupling can favor accumulation of inflammatory mediators or misfolded proteins, while inflammation and protein pathology may further impair vascular compliance, astrocyte polarization, AQP4 organization, and barrier integrity (Hauglund et al., 2025; Huang H. et al., 2024; Ishida et al., 2022). This bidirectional relationship suggests that neurodegeneration should not be framed as a purely hydrodynamic disorder, but as a coupled mechanical–vascular–glial–immune failure mode.

Therapeutically, restoring effective pulsatile coupling and improving perivascular exchange are attractive but still testable goals. Potential approaches include optimizing vascular compliance, modulating respiratory or posture-dependent CSF dynamics, reducing neuroinflammation, and targeting mechanosensitive pathways that influence NVU or astrocytic responses. Future studies should pair imaging-derived coupling metrics, such as blood-to-CSF pulsatile transfer and periarterial waveform features, with molecular and cellular readouts including AQP4 organization, BBB permeability, inflammatory markers, and protein-clearance indicators. Such paired measurements are essential for determining whether an intervention truly improves exchange/clearance biology rather than merely altering bulk CSF motion.

### 5.3 Aging: stiffening, altered mechanosensitivity, and multi-barrier vulnerability

Aging reshapes the mechanical milieu of the CNS through vascular stiffening, extracellular matrix remodeling, and altered craniospinal compliance, thereby changing how pulsatile energy is buffered, transmitted, and distributed across CSF-adjacent compartments (Kasprowicz et al., 2025; Wright et al., 2024; Zheng et al., 2024). When buffering capacity declines, mechanical load may be redistributed toward CSF-facing barriers and interfaces, increasing exposure to pressure/flow oscillations, deformation, and altered near-wall shear histories (Gholampour, 2023). In parallel, matrix remodeling can retune cellular baseline tension and mechanotransduction set-points, making barrier phenotypes more responsive—or more vulnerable—to mechanical cues that may otherwise be tolerated in younger tissues (Hansen et al., 2024a; Hitpass Romero et al., 2025). Thus, aging can be viewed as a mechanical endotype modifier that alters both exposure and cellular mechanosensitivity.

This vulnerability is likely multi-compartmental. At the perivascular/NVU interface, age-related vascular stiffening and reduced pulsatile coupling may impair CSF–ISF exchange and clearance-related metrics. Human imaging studies associate aging with altered perivascular clearance proxies and cognitive outcomes, supporting the plausibility of impaired exchange in aging, but these associations remain indirect and do not establish causality (Wang et al., 2023). At the outflow end, age-related meningeal extracellular matrix remodeling has been linked to reduced meningeal lymphatic function and diminished CSF clearance capacity (Hitpass Romero et al., 2025). These observations suggest that aging may simultaneously affect pulsatile exchange, barrier mechanosensitivity, and outflow efficiency rather than acting through a single CSF pathway.

Preclinical intervention data should be interpreted separately from human observational evidence. In mice, non-invasive modulation of meningeal lymphatic function has been reported to ameliorate aging- and Alzheimer’s disease–associated pathology and cognitive phenotypes, suggesting that the outflow compartment may be therapeutically tractable (Jin et al., 2025; Wang M. et al., 2024). However, translation to humans requires caution because lymphatic anatomy, disease timescale, vascular comorbidity, and measurement approaches differ across species.

A critical appraisal is therefore necessary. Many human clearance proxies are sensitive to factors that co-vary with age, including sleep quality, small-vessel disease, cardiovascular stiffness, respiratory pattern, and posture. Aging may also produce heterogeneous mechanical endotypes that are obscured by pooled age-group analyses. Mechanomedicine approaches should therefore combine CSF pulsatility/coupling measures, vascular compliance proxies, outflow metrics, and barrier-relevant phenotyping, rather than treating age as a demographic covariate alone.

### 5.4 Meningeal outflow failure: lymphatic dysfunction and neuroinflammatory amplification

Meningeal outflow is increasingly recognized as a coupled clearance-and-immune interface. In addition to classical arachnoid granulation–venous drainage routes, meningeal lymphatic pathways along dural sinuses contribute to solute removal and immune surveillance, thereby linking CSF clearance to dural and cervical lymph node immune circuits (Jacob et al., 2022; Matrongolo et al., 2023). When outflow efficiency declines because of aging, neuroinflammation, venous pressure elevation, or altered pressure gradients, CSF-adjacent solute retention and immune activation may be amplified, with downstream effects on parenchymal homeostasis.

From a mechanomedicine perspective, meningeal outflow can be viewed as an operating range constrained by pressure gradients, venous pressure, meningeal stiffness, and dural extracellular matrix (ECM) composition. Age-related meningeal ECM remodeling has been associated with reduced lymphatic function and impaired CSF clearance capacity, suggesting that tissue mechanical state may influence the efficiency of drainage and immune trafficking (Hitpass Romero et al., 2025). Blood pressure lowering has also been linked to enhanced CSF efflux through lymphatic routes, supporting the concept that systemic vascular and pressure states may modulate outflow capacity (Jukkola et al., 2024).

However, the evidence base should be interpreted with attention to species and methodology. Human studies support the existence and clinical relevance of meningeal lymphatic drainage circuits, whereas many mechanistic intervention data derive from mouse or other preclinical models (Hitpass Romero et al., 2025; Jacob et al., 2022; Matrongolo et al., 2023). Therefore, therapeutic extrapolation should remain cautious: interventions that improve lymphatic structure or drainage in animals should be considered hypothesis-generating unless paired with validated human outflow, immune, and clinical endpoints.

Meningeal outflow failure may amplify neuroinflammation through a feed-forward loop. Reduced drainage can limit clearance of solutes and inflammatory mediators, while dural immune activation, ECM remodeling, or elevated venous pressure may further restrict outflow and alter CSF pressure–flow coupling. Meningeal lymphatic–microglial signaling further supports a mechanistic link between impaired drainage, CNS immune tone, and neural function (Kim et al., 2025). This feedback is relevant to disorders in which clearance failure and inflammation coexist, including aging-related cognitive decline, Alzheimer’s disease, hydrocephalus, and post-traumatic states (Hitpass Romero et al., 2025; Wang M. et al., 2024).

A mechanomedicine priority is therefore to define outflow-targeted interventions using paired mechanical, clearance, and immune readouts. Useful endpoints include quantitative CSF efflux metrics, dural or meningeal stiffness measures, venous pressure or pressure-gradient proxies, lymphatic vessel morphology and coverage, cervical lymph node drainage, inflammatory cytokine profiles, immune-cell trafficking, and imaging-derived clearance or coupling metrics. Such readouts can determine whether pressure-modulating, posture/respiratory, pharmacological, or lymphatic-targeted strategies produce measurable changes in outflow function and neuroinflammatory state.

### 5.5 Trauma and inflammation: acute mechanical disruption and inflammatory amplification

Traumatic brain injury (TBI) introduces an abrupt mechanical perturbation to the CSF–barrier system, rapidly altering intracranial pressure dynamics, flow regimes, tissue deformation, and effective compliance (Solár et al., 2020; Uryga et al., 2023). These acute forces can overload CSF-facing barriers and NVU-associated interfaces, destabilizing the BBB, BCSFB, ependymal surface, and perivascular exchange routes. In the early phase, rapid pressure and shear changes may disrupt junctional integrity and increase barrier permeability, enabling plasma protein leakage, hemorrhagic components, and inflammatory mediators to enter normally protected compartments (Munoz-Ballester et al., 2022).

The secondary phase is characterized by inflammatory amplification superimposed on the initial mechanical insult. Cytokine production, immune-cell infiltration, and glial activation can further weaken barrier integrity and alter perivascular or CSF–tissue exchange (Alam et al., 2020; Nelles and Hazrati, 2023). In parallel, ependymal injury, glycocalyx remodeling, and altered tissue stiffness may disturb CSF circulation and clearance, creating a feedback loop in which mechanical disruption and inflammation reinforce each other (Duy et al., 2024; Iida et al., 2025).

This coupling is clinically important because post-traumatic CSF dysfunction may evolve toward hydrocephalus-like phenotypes, impaired clearance, or longer-term neurodegenerative vulnerability. However, these trajectories should not be interpreted as consequences of bulk CSF flow change alone. Instead, TBI should be viewed as a time-dependent mechanical–immune failure mode in which pressure waveform abnormalities, local deformation, barrier leakage, inflammatory tone, and clearance impairment may evolve together.

The mechanomedicine implication is that acute and subacute TBI studies should track both the mechanical trajectory and the immune/barrier trajectory. Relevant exposure metrics include ICP waveform features, pressure–volume or compliance proxies, CSF flow–volume coupling, and near-wall or model-estimated deformation. These should be paired with barrier and immune endpoints, including BBB/BCSFB permeability, tight-junction markers, plasma protein extravasation, cytokine profiles, immune-cell trafficking, ependymal integrity, glycocalyx state, and perivascular exchange markers (Alam et al., 2020; Iida et al., 2025; Munoz-Ballester et al., 2022; Nelles and Hazrati, 2023; Uryga et al., 2023). Such paired measurements can help distinguish primary mechanosensing from secondary injury responses and evaluate whether interventions targeting pressure, flow, inflammation, or mechanotransduction reduce the risk of chronic post-traumatic CSF–barrier dysfunction.

## 6 Toward mechanomedicine: targeting CSF–barrier mechanics with measurable outcomes

**FIGURE 4:**
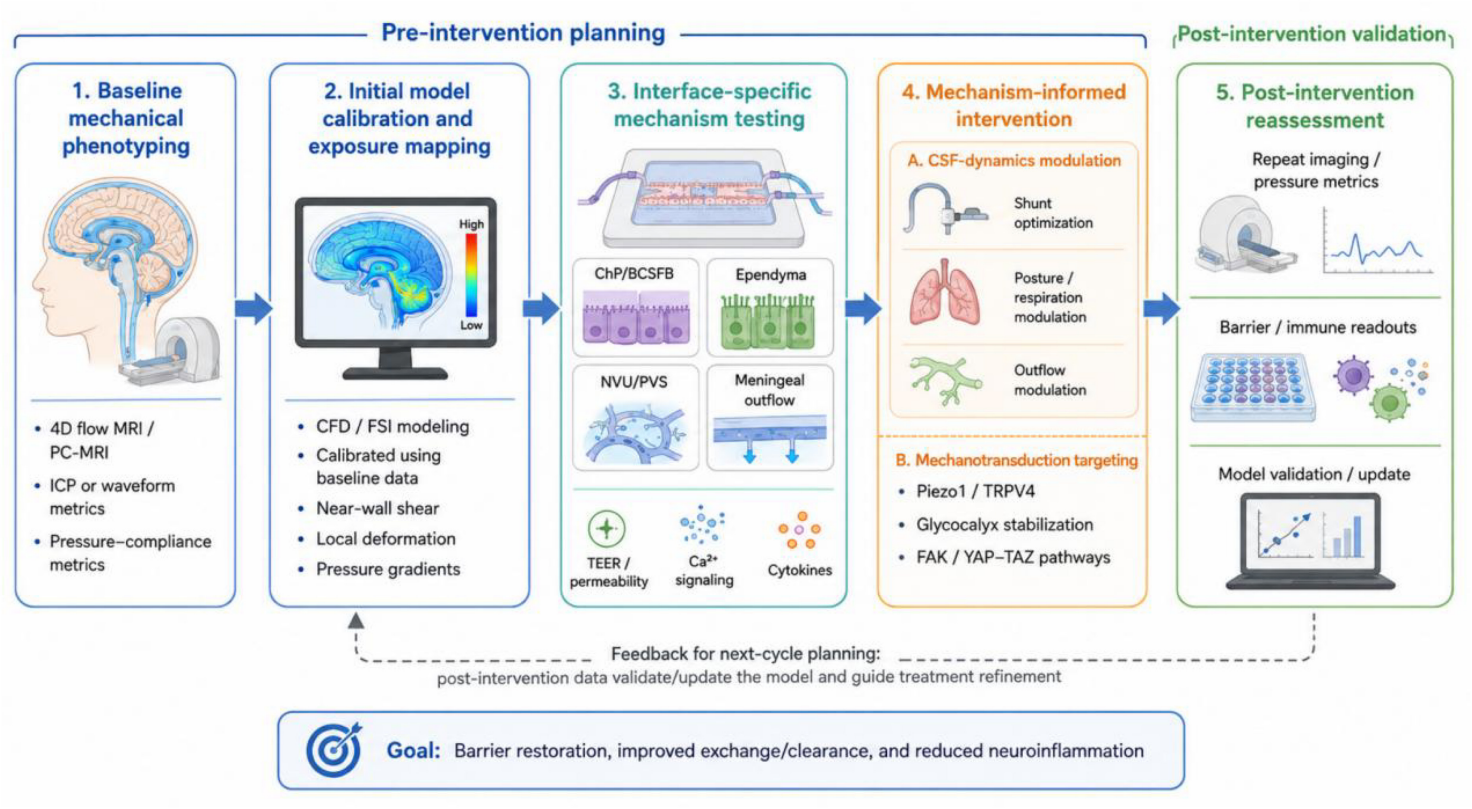
A closed-loop translational pipeline for CSF mechanomedicine. This schematic illustrates a staged workflow linking patient-scale mechanical phenotyping to model-based exposure estimation, interface-specific mechanism testing, mechanism-informed intervention, and post-intervention reassessment. Steps 1-4 represent pre-intervention planning. (1) Baseline mechanical phenotyping uses 4D flow MRI, phase-contrast MRI, ICP or waveform metrics, and pressure-compliance metrics to define patient-specific CSF mechanical states. (2) Initial model calibration and exposure mapping use baseline data to constrain CFD/FSI models and estimate difficult-to-measure mechanical exposures, including near-wall shear, local deformation, and pressure gradients. (3) Interface-specific mechanism testing translates these estimated exposures into experimentally testable conditions at the choroid plexus/blood-CSF barrier, ependymal, neurovascular/perivascular, and meningeal outflow interfaces, with readouts such as TEER/permeability, Ca^2 +^ signaling, and cytokine responses. (4) Mechanism-informed intervention selection may include CSF-dynamics modulation, such as shunt optimization, posture or respiration modulation, and outflow modulation, as well as targeted mechanotransduction strategies involving candidate nodes such as Piezo1/TRPV4, the glycocalyx, FAK, and YAP-TAZ pathways. Step 5 represents post-intervention validation, in which repeat imaging and pressure metrics are paired with barrier and immune readouts to determine whether the intended mechanical and biological effects were achieved. These post-intervention data do not modify the imaging modality itself; rather, they validate or update model parameters and guide next-cycle treatment refinement. The overall goal is barrier restoration, improved CSF exchange or clearance, and reduced neuroinflammatory amplification. BCSFB, blood-CSF barrier; CFD, computational fluid dynamics; ChP, choroid plexus; CSF, cerebrospinal fluid; FAK, focal adhesion kinase; FSI, fluid-structure interaction; ICP, intracranial pressure; MRI, magnetic resonance imaging; NVU, neurovascular unit; PC-MRI, phase-contrast MRI; PVS, perivascular space; TEER, transepithelial/transendothelial electrical resistance.

Cerebrospinal fluid mechanomedicine aims to identify maladaptive mechanical exposures, vulnerable CSF-facing interfaces, and measurable barrier endpoints rather than simply normalizing CSF flow. This requires a closed-loop workflow linking patient-specific imaging, model-based exposure estimation, in vitro mechanism testing, and post-intervention reassessment. Relevant endpoints should combine mechanical metrics with biological readouts such as permeability, junctional integrity, mechanosensor activation, immune markers, and clearance-related coupling. The proposed closed-loop workflow linking patient-specific imaging, model-based exposure estimation, interface-specific mechanism testing, intervention selection, and post-intervention reassessment is summarized in Figure 4.

### 6.1 Modulating CSF dynamics to restore barrier phenotypes

Modulating CSF dynamics is a central intervention axis for disorders in which pressure, pulsatility, compliance, or outflow abnormalities impose maladaptive loading on CSF-facing barriers. Rather than treating CSF flow as a single therapeutic target, intervention design should specify the mechanical exposure to be changed—such as excessive pulse transmission, reduced effective pulsatility, impaired outflow, or abnormal pressure–volume coupling—and the barrier phenotype expected to improve.

In hydrocephalus and related CSF circulation disorders, shunt-related optimization may alter pressure–flow relationships in selected patients (Capel et al., 2023), whereas ChP/BCSFB-targeted modulation may be relevant when hypersecretion or barrier dysfunction contributes to CSF imbalance (Wang et al., 2025). Strategies that improve compliance-related buffering, reduce abnormal ICP waveform transmission, or physiologically modulate respiration/posture may further reshape CSF pulsatility, pressure gradients, and outflow efficiency (Hladky and Barrand, 2024; Lee and Baik, 2024; Lim et al., 2025; Muccio et al., 2021). These approaches should be assessed not only by ventricular size or symptoms, but also by exposure-linked readouts, including ICP waveform features, CSF flow–volume coupling, compliance proxies, model-estimated loading, ependymal/BCSFB integrity, permeability, and inflammatory markers.

For mechanomedicine translation, dynamic interventions should be embedded in a closed-loop workflow. Baseline imaging and waveform analysis should define patient-specific mechanical exposure; computational or in vitro models should estimate the cellular loading regime and nominate intervention targets; and post-intervention imaging, pressure metrics, and barrier/immune readouts should determine whether the intended mechanical and biological effects were achieved. This framework can help distinguish interventions that merely alter bulk CSF motion from those that measurably improve CSF–barrier coupling and barrier phenotype.

### 6.2 Targeting mechanosensors and downstream effectors at CSF interfaces

Targeting mechanosensors and downstream effectors provides a complementary strategy to direct modulation of CSF dynamics. Rather than treating mechanosensors as isolated drug targets, CSF mechanomedicine should define which mechanical cue activates which sensor in which interface, and whether target engagement improves a measurable barrier phenotype. Candidate targets include mechanically responsive ion or transport channels, surface and matrix-associated mechanosensors, cytoskeletal–junctional regulators, and nuclear mechanotransduction pathways. At present, many of these targets should be considered mechanistic or preclinical candidates rather than established clinical therapies.

Among these candidates, Piezo1 and TRPV4 remain important testable nodes because they can couple shear, stretch, membrane tension, or pressure-related loading to Ca^2 +^ signaling, cytoskeletal remodeling, inflammatory activation, and permeability changes (An et al., 2025; Cibelli et al., 2024; Hansen et al., 2024b; Hochstetler et al., 2020; Pabst et al., 2026). In hydrocephalus and inflammatory CSF disorders, TRPV4-related signaling has been linked to CSF accumulation and barrier-relevant responses, supporting the rationale for pathway-specific modulation in selected contexts (Hansen et al., 2024b; Hochstetler et al., 2020; Toft-Bertelsen et al., 2022). However, channel gain is state dependent; therefore, studies should stratify or control for baseline inflammatory tone when testing Piezo1/TRPV4-directed interventions (Toft-Bertelsen et al., 2022; Yu et al., 2023).

Beyond Piezo1/TRPV4, additional candidate modules include TREK-1/TRAAK K2P channels, ENaC-related sodium transport, glycocalyx-mediated surface mechanosensing, and ECM–integrin–FAK signaling (Baldin et al., 2020; Brohawn et al., 2014; Hollósi et al., 2021; Sun et al., 2016; Tarbell and Pahakis, 2006). The strength of evidence differs across ChP, ependymal, NVU-associated, and meningeal interfaces. For example, TREK-1/TRAAK and ENaC may provide candidate links between membrane tension, ionic homeostasis, and fluid/solute handling, whereas glycocalyx or ECM–FAK pathways may tune the mechanical set-point of barrier cells under aging, inflammation, hydrocephalus, or trauma.

Downstream effectors may be equally important intervention points. Ca^2 +^ signaling, actomyosin tension, tight/adherens-junction remodeling, FAK activation, and YAP/TAZ nuclear localization can convert transient mechanical exposures into sustained barrier states (Du et al., 2024; Gong et al., 2021; Hollósi et al., 2021; Peng et al., 2025; Sun et al., 2024). Modulating these downstream nodes may be useful when direct channel targeting is not feasible or when multiple upstream sensors converge on shared permeability, inflammatory, or repair pathways.

For translation, each candidate therapy should be paired with a predefined intermediate phenotype and a functional endpoint. Useful target-engagement readouts include Ca^2 +^ dynamics, electrophysiological activity, transporter/channel expression, glycocalyx integrity, FAK activation, cytoskeletal organization, junctional protein localization, YAP/TAZ localization, TEER, tracer permeability, cytokine profiles, and imaging-derived CSF coupling metrics. This design can distinguish true mechanomodulation from non-specific anti-inflammatory or symptomatic effects and can identify whether therapeutic benefit tracks with the intended mechanical exposure.

### 6.3 Experimental pipelines and translational considerations

Translating CSF mechanomedicine requires pipelines that connect patient-scale measurements to cell-scale mechanisms and intervention-linked endpoints. A practical workflow begins with *in vivo* phenotyping using low-velocity 4D flow MRI, phase-contrast MRI, and waveform analysis to quantify CSF velocity, pulsatility, pressure–flow coupling, and compartment-level flow regimes (El-Sayed Sakr et al., 2023; Karki et al., 2024; Liu et al., 2025; Vikner et al., 2024; Whitley et al., 2024). These measurements define patient-specific boundary conditions but should not be treated as direct measures of near-wall shear or cellular deformation.

Computational models, including CFD and fluid–structure interaction frameworks, can then estimate difficult-to-measure exposures such as near-wall shear, local deformation, pressure gradients, and sensitivity to wall motion or compliance assumptions (Hurd et al., 2023; Putluru et al., 2025). These model-derived exposures should be calibrated against imaging data and used to generate experimentally testable loading conditions rather than interpreted as standalone predictions.

*In vitro* and *ex vivo* platforms provide the next mechanistic layer. Microfluidic barrier models, organotypic cultures, and interface-specific systems can impose defined shear, stretch, pressure, or pulsatile waveforms on ChP/BCSFB, ependymal, endothelial/NVU, or meningeal-relevant cell systems (Katoh, 2023; Meng et al., 2022). These platforms should pair mechanical inputs with matched biological readouts, including TEER, tracer permeability, Ca^2 +^ dynamics, channel activity, junctional localization, glycocalyx integrity, FAK/YAP–TAZ activity, cytokine profiles, and transport markers.

For translation, the pipeline should operate as a closed loop rather than a one-way sequence. Baseline imaging and waveform metrics define the mechanical phenotype; models estimate cell-level exposure; in vitro systems test candidate mechanisms and interventions; and post-intervention imaging, pressure metrics, and barrier/immune readouts determine whether the intended mechanical and biological effects were achieved. These results should then recalibrate the model and guide the next clinical or experimental decision.

Several considerations are essential for implementation. Mechanical metrics should be reported with acquisition parameters, anatomical region, posture or physiological state, and modeling assumptions. Biological readouts should be interface-specific, because ChP epithelium, ventricular ependyma, NVU-associated cells, and meningeal outflow structures differ in architecture and mechanosensitivity. Finally, interventions should be evaluated against pre-specified exposure-linked endpoints, allowing responders to be stratified by mechanical phenotype rather than by diagnosis alone.

## 7 Conclusion and future perspectives

Cerebrospinal fluid mechanics is an active regulator of CNS barrier and interface biology. Along the production-to-outflow axis, shear stress, pulsatile forcing, pressure–compliance relationships, and tissue stiffness may shape the phenotypes of the ChP/BCSFB, ventricular ependyma, NVU-associated perivascular pathways, and meningeal outflow routes through interface-specific mechanotransduction programs.

Disrupted CSF–barrier coupling can be interpreted as overlapping mechanical failure modes rather than a single flow abnormality. Hydrocephalus, neurodegeneration, aging, meningeal outflow dysfunction, and trauma each involve distinct combinations of altered mechanical exposure, barrier vulnerability, impaired exchange or clearance, and inflammatory amplification.

Future work should prioritize standardized and mechanistically interpretable measurements of CSF mechanics and barrier state. Key needs include validated imaging metrics, calibrated computational models for near-wall exposure estimation, experimentally controlled barrier platforms, and paired biological readouts such as permeability, junctional integrity, ciliary function, glycocalyx state, mechanosensor activation, and immune markers.

A practical mechanomedicine roadmap is to define patient-relevant mechanical exposures, map them to interface-specific sensors and intermediate phenotypes, and test interventions against pre-specified mechanical and biological endpoints. Closed-loop pipelines linking human imaging, model-based exposure estimation, experimental validation, and post-intervention reassessment may support more precise strategies to stabilize barriers, improve exchange or clearance, and limit neuroinflammatory amplification in CSF-related neurological disorders. For clinicians, this framework encourages assessment of CSF-related disorders through both fluid-dynamic changes and barrier-centered biological outcomes.
